# Tumor-associated macrophages and CD8+ T cells: dual players in the pathogenesis of HBV-related HCC

**DOI:** 10.3389/fimmu.2024.1472430

**Published:** 2024-10-10

**Authors:** Muhammad Naveed Khan, Binli Mao, Juan Hu, Mengjia Shi, Shunyao Wang, Adeel Ur Rehman, Xiaosong Li

**Affiliations:** ^1^ Clinical Molecular Medicine Testing Center, The First Affiliated Hospital of Chongqing Medical University, Chongqing, China; ^2^ Western (Chongqing) Collaborative Innovation Center for Intelligent Diagnostics and Digital Medicine, Chongqing, China; ^3^ Department of Blood Transfusion, The First Affiliated Hospital of Chongqing Medical University, Chongqing, China; ^4^ Department of Clinical Laboratory Medicine, Suining Central Hospital, Suining, Sichuan, China

**Keywords:** hepatitis B virus, CD8+ T cell, TAMs-like macrophage, HBV-related HCC, pathogenesis, immunology

## Abstract

HBV infection is a key risk factor for the development and progression of hepatocellular carcinoma (HCC), a highly invasive tumor, and is characterized by its persistent immunosuppressive microenvironment. This review provides an in-depth analysis of HBV-related HCC and explores the interactions between neutrophils, natural killer cells, and dendritic cells, examining their roles in regulating tumor-associated macrophages and CD8+ T cells and shaping the tumor microenvironment. Two critical players in the immunosuppressive milieu of HBV-related HCC are CD8+ T cells and tumor-associated macrophages (TAMs). The study explores how TAMs, initially recruited to combat infection, transform, adopting a tumor-promoting phenotype, turning against the body, promoting tumor cell proliferation, suppressing anti-tumor immunity, and assisting in the spread of cancer. Meanwhile, CD8+ T cells, crucial for controlling HBV infection, become dysfunctional and exhausted in response to persistent chronic viral inflammation. The review then dissects how TAMs manipulate this immune response, further depleting CD8+ T cell functions through mechanisms like arginine deprivation and creating hypoxic environments that lead to exhaustion. Finally, it explores the challenges and promising therapeutic avenues that target TAMs and CD8+ T cells, either separately or in combination with antiviral therapy and personalized medicine approaches, offering hope for improved outcomes in HBV-related HCC.

## 1 Introduction

Hepatocellular carcinoma (HCC) is the most prevalent cause of over 80% of primary liver cancers, and liver cancer is the sixth most frequent cancer worldwide in terms of cancer-related mortality (1, 2). Chronic hepatitis B virus (HBV) infection is a leading cause of liver cirrhosis, a condition that significantly increases the risk of developing HCC. In 2019, World Health Organization (WHO) reported a global prevalence of 296 million individuals with chronic HBV infection. Alarmingly, only 30.4 million individuals were aware of their hepatitis B status, and approximately 1.5 million people experienced new infections of chronic hepatitis B virus (3). HBV infection induces chronic liver injury, which initiates reparative mechanisms having the aim of restoring the structure and function of the liver (4). Nevertheless, the presence of chronic inflammation may result in ongoing regenerative repair of hepatocytes, hence contributing a role in the initiation and progression of HCC (5). In the tumor microenvironment (TME) of HBV-related HCC, various components, including immune cells, neoplastic cells, blood vessel cells, tumor-associated fibroblasts, and extracellular matrix, play a pivotal role in tumor (6). Understanding cancer progression requires a knowledge of immune cell interplay within the tumor microenvironment (TME), particularly with CD8+ T cells and tumor-associated macrophages (TAMs).

The tumor microenvironment, consisting of immune cells such as TAM-like macrophage, CD8+ T cells, neutrophils, NK cells, and DCs and non-immune cells such as stromal cells, endothelial cells, cytokines, and ECM, plays a key role in the initiation and progression of HCC (7, 8). Research highlights the significant infiltration of immune cells such as TAMs and CD8+ T cells in liver cancer, particularly in HBV-related HCC, correlating strongly with negative patient prognosis (9). The liver, compared to other organs, hosts a higher percentage of macrophages, including Kupffer cells (KCs), which are tissue-resident macrophages critical for maintaining homeostasis (10). TAMs constitute a pivotal component of the TME, actively contributing to cancer progression and metastatic dissemination (11). The multifaceted roles of TAMs in HCC have garnered significant attention, encompassing immunosuppressive function, facilitation of tumor invasion and metastasis, pro-angiogenic activity, induction of epithelial-mesenchymal transition (EMT), and promotion of stemness (12). A comprehensive understanding of these diverse TAM functions is crucial for the development of effective therapeutic strategies in HCC. Therapeutic targeting of TAMs holds promise for the development of novel treatment strategies in HCC, presenting an exciting area of burgeoning investigation (13). In addition to TAMs, CD8+ T cells also play a crucial role in the immune microenvironment of HBV-related HCC (14, 15). Interactions among tumor cells, TAMs, CD8+ T cells, and other immune components affect key aspects such as proliferation, invasion, migration, liver fibrosis, and immune killing of tumor cells through the secretion of cytokines and exosomes and alterations in the expression of related proteins (16). The intricate mechanisms and signaling pathways involved in the interplay between TAMs, CD8+ T cells, and other immune cells in HBV-related HCC underscore the complexity of the disease progression (17). This review article provides the interplay between TAMs and CD8+ T cells specifically contribute to the pathogenesis, progression, and clinical outcomes of HBV-related HCC, and what therapeutic strategies, both current and emerging, target these immune components, considering their limitations and potential for improving patient outcomes.

## 2 Crosstalk of immune cells with TAMs and CD8+ T cells

### 2.1 Immune cells interaction with TAMs and CD8+ T Cells

**Figure 1:**
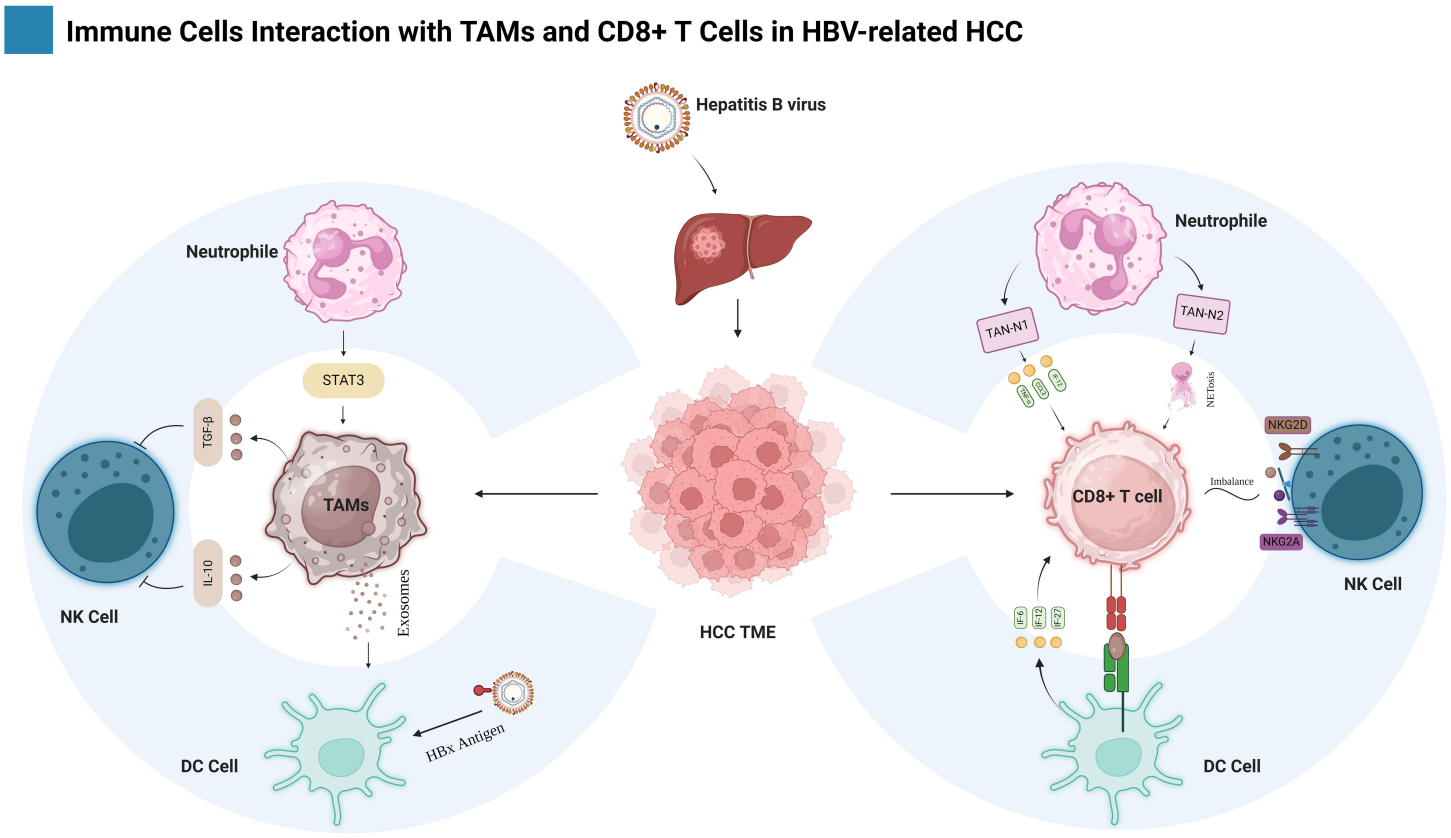
Shows the intricate interaction among different populations of immune cells in the context of HBV-related HCC. The primary emphasis is tumor-associated macrophages (TAMs) and CD8+ T cells as well as their interactions with other immune cells, such as Neutrophils, Natural killer (NK) cells and Dendritic cells (DCs). The figure is created with BioRender.com.

HBV infection can significantly impair the immune response, creating a conducive environment for HCC through various mechanisms involving CD8+ T cells and TAMs (18). HCC is characterized by a complex interplay between viral factors, tumor cells, and the host immune response. In HBV-related HCC, Tregs have been shown to enhance CD8+ T cell exhaustion, contributing to persistent viral infection and chronic inflammation (19). Additionally, Chronic HBV infection results in the accumulation of exhausted CD8+ T cells (Tex), characterized by high expression of inhibitory receptors like PD-1, leading to impaired anti-tumor immunity (18, 20). Furthermore, CD3+C1q+ TAMs interact with CD8+ T cells, influencing their function through metabolic reprogramming, which can further suppress T cell activity in the tumor microenvironment (TME) (21). The interplay between Tregs, exhausted CD8+ T cells, and TAMs fosters an immunosuppressive TME, facilitating HCC development and progression A hallmark of HBV-HCC is the development of immune dysfunction, which significantly contributes to tumor progression. This immune dysfunction involves alterations in various immune cell populations, including macrophages and CD8 T cells as shown in **Figure 1**.

Macrophages, key players in both innate and adaptive immunity, are profoundly affected by HBV infection. In the context of HBV-related HCC, macrophages often adopt a tumor-promoting phenotype, known as tumor-associated macrophages (TAMs) (6). TAMs can suppress anti-tumor immune responses via a variety of mechanisms, including immunosuppressive cytokine release, angiogenesis promotion, and T cell function inhibition. In addition, HBV can directly infect macrophages, resulting in their dysfunction and potentially contributing to an open environment for tumor growth (22).

CD8+ T cells, which are cytotoxic lymphocytes that eradicate virus-infected and tumor cells, are also markedly reduced in HBV-related HCC. HBV can resist CD8 T cell recognition in a variety of ways, including downregulating MHC class I expression on infected cells and generating viral proteins that inhibit T cell activation (23). Furthermore, HBV infection creates an immunosuppressive milieu that can impair CD8 T cell activity, restricting their ability to efficiently target and eliminate tumor cells (14).

#### 2.1.1 Neutrophil interactions with TAMs and CD8+ T cells

Neutrophils, key players of the innate immune system, readily infiltrate tissues upon infection, damage, or cancer (24). The two main phenotypes of tumor-associated neutrophils (TANs) are antitumorigenic (TAN-N1) and protumorigenic (TAN-N2), arise from differential activation levels (mediated by TGF-β) rather than distinct molecular profiles (25). Neutrophils employ diverse mechanisms to combat cancer, including direct tumoricidal action via antibody-dependent cellular cytotoxicity (ADCC), and indirect orchestration of antitumor adaptive immune responses (26, 27). Neutrophils can interact with various immune cell types, including tumor-associated macrophages (TAMs) and CD8+ T cells, to influence the tumor microenvironment and anti-tumor immune responses.

In HBV-related HCC, TANs have a pro-tumor phenotype, promoting tumor growth and metastasis although interactions with TAMs create a supportive microenvironment for tumor cells by releasing factors that suppress anti-tumor immune responses and promote angiogenesis (28). Zhou et al. showed that the interaction between tumor-associated neutrophils (TANs) and tumor-associated macrophages (TAMs) promotes the proliferation and propagation of cancer cells by activating the STAT3 signaling pathway (29). Moreover, TAN-N1 indirectly regulates the recruitment and activation of CD8+ T cells by producing chemokines and pro-inflammatory cytokines such as CCL3, CCL9, CXCL10, TNF-α, and IL-12, thereby contributing to the restriction of tumor growth (30). Additionally, N1 TAN-derived cytokines stimulate T cell proliferation and differentiation, promoting an antitumor Th1 response.

On the other hand, Protumorigenic TAN-N2 produces decondensed chromatin structures embedded with granular proteins, called neutrophil extracellular traps (NETs), which are known to contribute to tumor development (31). Additionally, NETs have been shown to induce tumor metastasis by promoting the migration of cancer cells and down-regulating tight junction molecules on endothelial cells (32). The priming of neutrophils for NETosis by tumors has been found to favor tumor growth (33). Nevertheless, a higher number of CD66b+ neutrophils found around tumors is associated with a lower chance of survival in patients with hepatocellular carcinoma (HCC). These findings collectively suggest that NETs and CD66b+ neutrophils play a significant role in the progression of HCC (34). These interactions contribute to the immunosuppressive microenvironment often observed in HBV-related HCC, hindering the ability of the immune system to effectively eliminate tumor cells.

#### 2.1.2 NK cell interactions with TAMs and CD8+ T cells

Natural killer cells are distinctive cytotoxic lymphocytes that play a vital role in fighting tumors and infections (35). Human NK cells comprise approximately 15% of total lymphocytes and the frequency of circulating NK cells is reduced in the blood of Hepatitis B and C viral infections, while their frequency is elevated within the liver (36, 37). NK cells can interact with TAMs and CD8+ T cells, influencing the tumor microenvironment and immune responses.

NK cells can be negatively regulated by TAMs through various mechanisms. TAMs can release immunosuppressive factors, such as TGF-β and IL-10, which can inhibit NK cell function (38). Additionally, TAMs can express ligands for inhibitory receptors on NK cells, such as NKG2D and PD-L1, leading to NK cell dysfunction. Conversely, NK cells can also influence TAMs (39). Furthermore, TAMs phenotype and function can be altered by the cytokines and chemokines produced by activated NK cells, possibly leading to a more anti-tumor phenotype (40). On the other hand, CD8+ T cell and NK cell interaction, the expression levels of NKG2A in tumor-infiltrating tissues and peripheral blood were similar for NK cells, CD8 T cells showed a large difference in expression levels of NKG2A in tumor-infiltrating tissues and peripheral blood, where NKG2A is a constant companion, CD8+ T cells play a game of hide-and-seek with this inhibitory receptor. In healthy individuals, it’s practically invisible, but in the battlegrounds of tumors and chronic infections, NKG2A emerges as a major player (41, 42). Moreover, in HBV-related HCC the imbalance between inhibitory receptor NKG2A and activating receptor NKG2D on NK cells is associated with NK cell immunosuppression and tumor progression (43). The NKG2A/NKG2D ratio on NK cells can predict tumor progression and is inversely correlated with progression-free survival in HCC patients (43).

#### 2.1.3 DC cell interactions with TAMs and CD8+ T cells

Dendritic cells (DCs) serve as crucial intermediaries between innate and adaptive immunity by orchestrating antigen presentation, a process that ultimately leads to T cell activation and differentiation (44). In HBV-related HCC, DCs can interact with TAMs and CD8+ T cells to influence the tumor microenvironment and anti-tumor immune responses. Unlike macrophages, DCs exhibit migratory capacity and preferentially engage T cells within tissue-draining lymph nodes, although substantial DC-T cell interactions also occur in the liver (45, 46). Notably, the activation of CD8+ cytotoxic T cells hinges upon the prior activation of DCs by CD4+ T helper (Th) cells (47).

DCs play a crucial role on regulating immune responses in HBV-related HCC, particularly through their interactions with TAMs. Studies have shown that TAMs can release exosomes that transfer molecules to DCs, influencing their function. For instance, exosomes derived from TAMs can carry lncRNAs that stimulate tumor cell glycolysis, thereby enhancing tumor and further suppressing DC activity (48). Research indicates that TAMs help HCC patients evade immune responses by upregulating arginase, which cause an increase in proline production. This proline subsequently activates cancer-associated fibroblasts (CAFs), enhancing their expression of immune checkpoint molecules like PD-L1 and CTLA-4, thereby promoting immune suppression (49). In contrast, DCs pulsed with heat shock protein 70 and HBxAg have been shown to increase their maturation and stimulate robust antitumor T-cell responses, indicating a potential therapeutic approach to counteract the immunosuppressive environment created by TAMs and CAFs (50). Furthermore, A novel dendritic-cell-targeting DNA vaccine for hepatitis B, capable of inducing robust T cell and humoral immune responses, has demonstrated potent antiviral activity in HBV transgenic mice. Moreover, innovative strategies for generating dendritic cells from PBMCs, coupled with targeting HBV antigens, offer a promising strategy for developing effective immunotherapies against HCC (51). Thus, the interplay between DCs and TAMs is pivotal in shaping the immune landscape in HBV-related HCC, highlighting the need for targeted immunotherapeutic strategies.

DCs engage T cells through a complex interplay of antigen presentation, costimulation, and cytokine signaling. Initially, DCs must present the antigen to CD4+ T cells via MHC-II molecules and CD8+ T cells via MHC-I molecules (52). Costimulatory molecules from the immunoglobulin superfamily (CD80-CD86/CD28) and the TNF superfamily (CD40L/CD40, 4-1BBL/4-1BB, CD27/CD70, CD30L/CD30, and HVEM/LIGHT) play a critical role in the activation and stimulate the production of cytokines that promote CD8+ T cell expansion and differentiation (53). In addition to TCR/MHCp interactions, costimulatory pathways involving receptors like CD28 and ICOS on T cells and their ligands on DCs are crucial for T cell activation and differentiation (54). Furthermore, DCs secrete cytokines such as interleukin-6 (IL-6), IL-12, IL-23, IL-27, transforming growth factor-beta 1 (TGF-β1), retinaldehyde dehydrogenase (Raldh), and indoleamine 2,3-dioxygenase (IDO), which play important roles in shaping immune responses. Disruption of any mechanisms will lead to an erroneous adaptive response. As a result, the primary strategies by which cancer cells evade immune surveillance by disrupting the immunological synapse, often through the expression of inhibitory ligands that suppress T-cell activation (55). Hence, Cancer cells establish immune privilege by upregulating the expression of inhibitory ligands, such as PD-L1, CTLA-4, LAG-3, TIM-3, and GAL9, which engage their cognate receptors on T cells, effectively transmitting a “stop” signal that abrogates T cell activation and function (56). This suppression of the antitumor immune response facilitates tumor growth and evasion.

## 3 TAMs as an innate immune response against HBV-related HCC

### 3.1 TAMs origins and activation in HBV-related HCC

TAM origins are very complex and heterogeneous. It can originate from Kupffer cells (KCs), peripheral blood monocytes, and M-MDSCs. Tissue hepatic macrophages, often exemplified by Kupffer cells (57), have been shown to originate from two distinct embryonic sources: yolk sac-derived erythro-myeloid progenitors (EMPs) expressing the macrophage colony-stimulating factor 1 receptor (CSF1R), and EMPs arising within the fetal liver itself (58, 59). Kupffer cells (KCs), a tissue-resident macrophages, exhibit a complex duality in HCC, and contributing to anti-tumor immunity, they can also facilitate HCC progression (60, 61). Danger-associated molecular patterns (DAMPs) within HCC can engage the inflammatory program of KCs, leading to subsequent recruitment of immune cells to the liver. Notably, CCL2 production by tumor cells depletes resident embryonic KCs, paving the way for infiltration by monocyte-derived KCs and immature monocytes (M0) (62, 63). Inflammatory mediators secreted by cancer cells (such as chemokines and growth factors) within both primary and metastatic tumors drive their differentiation into TAMs, consequently promoting tumor progression (63).

Normally, Tissue macrophages and peripheral monocytes exhibit clear phenotypic and functional divergence. However, during inflammatory and neoplastic processes, peripheral monocytes derived from the bone marrow (BM) emerge as key contributors to the macrophage population, particularly TAMs, driven by a complex interplay of inflammatory and tumor-derived signals, these circulating monocytes undergo mobilization and infiltration into the TME, where they undergo further differentiation into tissue macrophage (64). Moreover, HCC fosters the differentiation of TAMs through Tim-3, a transmembrane protein with elevated expression in monocytes and TAMs. In HCC, this protein plays a pivotal role in activating and amplifying the pro-tumoral effects of TAMs (65). Chronic liver injury exhibits a strong association with the infiltration of M2-TAMs, the majority of which originate from CCR2-positive monocytes in bone marrow. The presence of CCR2-positive myeloid cells is critical for effective senescence surveillance, as evidenced by their depletion leading to HCC outgrowth (62). Monocyte recruitment to the TME is orchestrated by the CCL2/CCR2 axis, which differentiates into TAMs and polarizes towards an M2 phenotype, thereby actively contributing to HCC progression.

TAMs can be derived from myeloid-derived suppressor cells. MDSCs, derived from the bone marrow, can be classified into two subsets: granulocytic or polymorphonuclear (PMN-MDSCs) and monocytic (M-MDSCs) (66). PMN-MDSCs exhibit similarities to neutrophils, whereas M-MDSCs possess traits that are akin to monocytes (67). Tumor tissues contain a type of immune cells called M-MDSCs that have the ability to change and develop into either TAMs or inflammatory dendritic cells.

### 3.2 Macrophage polarization (M1 to M2)

**Figure 2:**
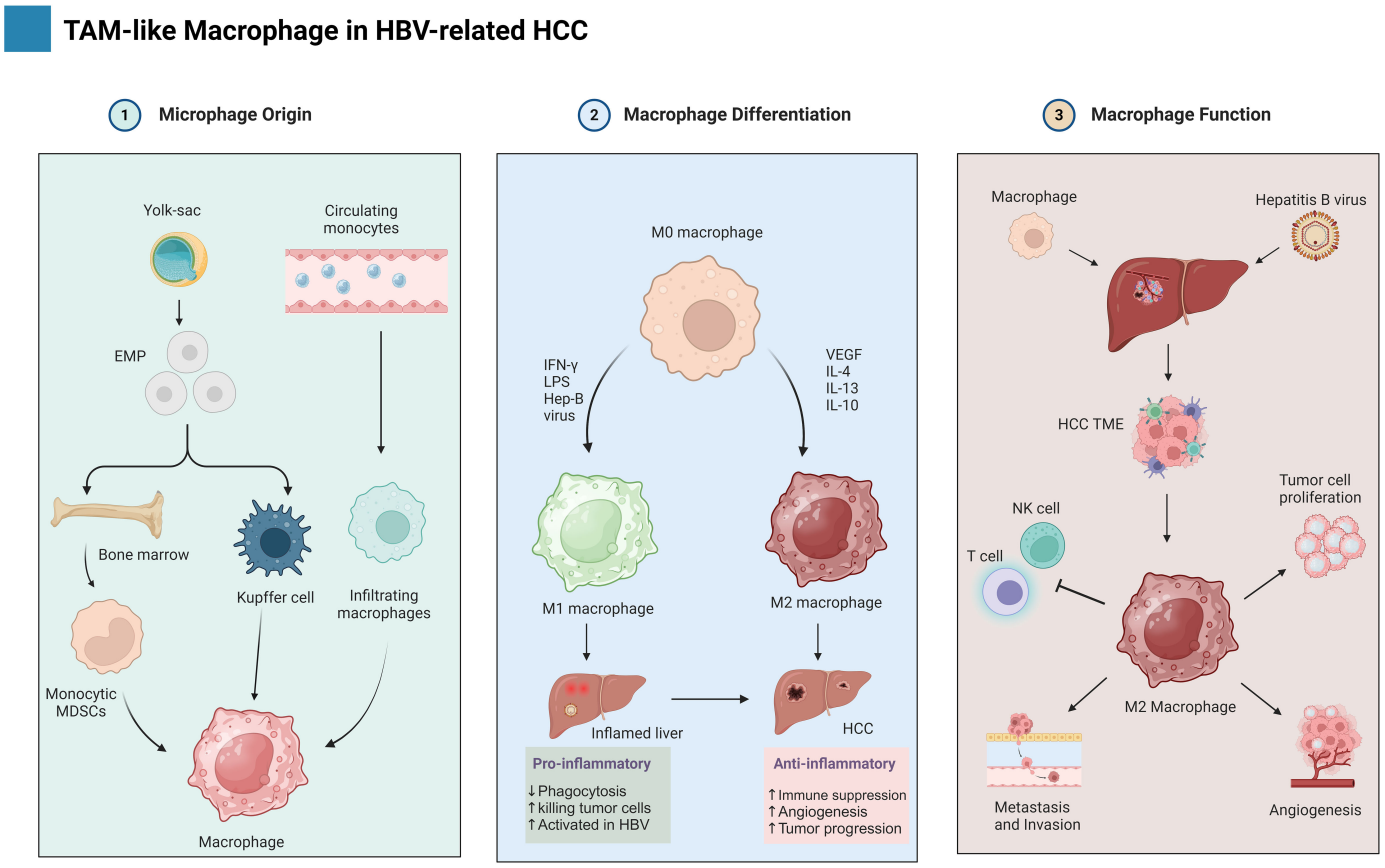
Shows the development of macrophages in HBV-HCC, showing their diverse origins and the mechanism of M1/M2 polarization, as well as their role in cancer. The figure is created with BioRender.com.

Macrophages have been categorized into two activation states: M1 macrophages with pro-inflammatory functions (68) and M2 macrophages with anti-inflammatory functions (69). Most TAMs display an M2-like phenotype, characterized by fusiform morphology (70) and macrophages acquire a pro-tumorigenic phenotype characterized by upregulated expression of genes like MMP14 (degrades extracellular matrix) (71), VEGF-A/D (promote angiogenesis), and MRC1/CD206 receptor (suppress antitumor immune response) (72–74). In HCC TME, enriched with cytokines and extracellular vesicles, various factors drive TAM polarization such as Wnt ligand secretion by tumor cells activates the Wnt/β-catenin pathway in macrophages, triggering β-catenin nuclear translocation and upregulating C-MYC transcription, thereby promoting M2-TAM polarization (75). IL-37 promotes the polarization of M2-TAMs toward M1-TAMs in HCC by inhibiting IL-6/STAT3 signaling (76). In HCC, elevated B7 homolog 3 (B7-H3) expression promotes TAM polarization towards the M2 phenotype via STAT6 signaling activation (77). Tumor-derived extracellular vesicles (EVs) containing miR4458H promote M2-like polarization of TAMs by upregulating arginase-1 (Arg1) expression (78). In HCC, high TREM1 expression characterizes M2 TAMs. Downregulating TREM1 induces their shift towards an M1 phenotype, primarily via PI3K/AKT/mTOR signaling pathway inhibition (79). Zip9 (Zinc/iron-regulated transporter-like protein) exhibits elevated expression on M2-polarized TAMs in HCC. This heightened expression promotes M2 polarization via STAT6 pathway activation, while concurrently suppressing M1 polarization through IκBα/β signaling inhibition (80). M1-TAMs exhibit higher ferrous iron content compared to their M2 counterparts. However, elevated transferrin expression in HCC cells restricts iron uptake by TAMs, promoting M2 polarization via HIF-1α upregulation (81). Moreover, despite transitioning towards an M2-like phenotype, retention of M1-associated transcriptomic signatures is frequently observed in macrophages within the HCC microenvironment (17). This intricate interplay influences tumor progression in diverse ways, potentially both promoting and inhibiting it as illustrated in **Figure 2**.

### 3.3 TAMs marker in HBV-related HCC

Tumor-associated macrophages (TAMs) play a crucial role in the progression of hepatocellular carcinoma (HCC). Several markers can be used to identify TAMs and characterize their phenotype within the tumor microenvironment. CD68, a pan-macrophage marker, is commonly used to identify TAMs in general. To assess the specific polarization of TAMs towards a pro-tumor phenotype, markers such as CD163, CD206, and Arg1 are employed (49, 82). These markers are associated with M2-polarized macrophages, which often exhibit immunosuppressive functions. Additionally, PD-L1, a checkpoint inhibitor, is expressed by some TAMs and can suppress anti-tumor immune responses (74). Furthermore, M2BPGi, IL-6, COMP, and Colony Stimulating Factor 1 Receptor (CSF1R) have been identified as potential markers for predicting HBV-related HCC, suggesting their involvement in the tumorigenic process (83, 84).

### 3.4 TAMs functions in tumor microenvironment

#### 3.4.1 Promoting tumor cell proliferation and survival

TAM roles are very complex in tumor cell proliferation and survival. In HBV-related HCC cells, TAMs promote tumor growth and survival by producing EGF and TGF- α/β, which induce a mesenchymal transition of epithelial and endothelial cells, alter differentiation and proliferation of immune cells, modulate matrix composition, and reprogram cell metabolism (85–87). However, preclinical investigations have found critical pathways regulating TAM infiltration and polarization throughout tumor growth. The lncRNA designated MAPKAPK5_AS1 (MAAS) exhibited marked elevation in M2 macrophages, a predominant subtype of TAMs observed in HCC-associated HBV infection. Furthermore, overexpression of MAAS demonstrably facilitated the proliferative capacity of HBV-infected HCC cells, both *in vitro* and *in vivo* (88). A further investigation revealed that PDZK1 is a gene associated with HBV infection that plays carcinogenic functions, potentially through augmenting PI3K-Akt, lipid acid consumption in tumor cells and TAMs, and Treg-induced immunosuppression (89). Recent studies have identified microRNAs (miRNAs), a class of small non-coding RNAs known to regulate gene expression at the post-transcriptional level, as potential mediators of tumor-promoting effects exerted by TAMs. MiR-146a-5p, enriched in exosomes derived from HCC cells, is a mediator of M2 TAM infiltration, contributing to T-cell exhaustion and HCC progression (90). These findings highlight the crucial role of miRNAs in the complex interplay between TAMs and tumor progression in HBV-related HCC, paving the way for miRNA-based interventions to modulate the tumor microenvironment.

#### 3.4.2 Suppressing anti-tumor immunity

In the intricate ecosystem of HBV-related HCC, TAMs emerge as double-edged swords, capable of initiating anti-tumor responses, their polarization towards the immunosuppressive M2 phenotype, heavily influenced by chronic HBV infection, orchestrates a potent suppression of anti-tumor immunity, fostering tumor progression (40). This intricate dance unfolds through a multifaceted interplay of signaling pathways, cytokine gradients, and metabolic reprogramming, ultimately creating a haven for HCC cells to thrive.

TAMs pose a significant obstacle to anti-tumor immunity through a multifaceted immunosuppressive arsenal. One key mechanism involves the PD-L1/PD-1 axis, where TAMs upregulate PD-L1 expression on their surface and tumor cells. This ligand-receptor interaction engages the PD-1 receptor on cytotoxic T lymphocytes (CTLs), triggering exhaustion and anergy, effectively shutting down their effector function and hindering their ability to recognize and eliminate tumor cells (91). Beyond immune checkpoint modulation, TAMs orchestrate metabolic subversion through arginase-1 activity, depleting essential amino acids like L-arginine from the tumor microenvironment (92). This creates a metabolically barren landscape that starves CTLs, hampering their proliferation and cytotoxic potential. Furthermore, TAMs actively recruit and activate CD4+ Tregs, potent suppressors of CTL activation and function. TAM-derived IL-10 and TGF-β promote Treg differentiation and expansion, while Treg-mediated CTLA-4 expression and IL-10 release further dampen the anti-tumor immune response (93). Additionally, TAMs downregulate MHC-I expression on tumor cells, hindering recognition and interaction with NK cells. Moreover, they suppress ligands like NKG2D ligands through IL-10 and TGF-β secretion, further impeding NK cell activation and cytotoxicity (94). TAMs compete with NK cells for glucose and L-arginine, creating a metabolically deprived environment that impairs NK cell function and cytotoxicity (95). Further investigations into the metabolic environment between TAMs and NK cells could identify novel therapeutic strategies to enhance NK cell function and improve anti-tumor immunity in cancer patients.

In HBV-related HCC, TAMs emerge as cunning conductors, orchestrating a multi-pronged assault on anti-tumor immunity. Firstly, TAMs release immunosuppressive cytokines like IL-10, TGF-β, and VEGF, dampening the overall immunological fervor and fostering a pro-tumorigenic microenvironment (96). This cytokine orchestrated symphony silences effector T cells and natural killer (NK) cells, while simultaneously promoting angiogenesis, creating a permissive environment for tumor growth (97). Secondly, TAMs recruit and polarize neutrophils toward a pro-tumorigenic N2 phenotype. These N2 neutrophils further suppress T and NK cell activity and contribute to angiogenesis, exacerbating the immunosuppressive microenvironment (98). Thirdly, the hepatitis B virus X protein (HBx) interacts with TAMs through the Toll-like receptor 4 (TLR4) pathway, activating the NF-κB signaling cascade and resulting in increased production of interleukin-10 (IL-10). This immunosuppressive cytokine further dampens T cell responses, reinforcing TAM dominance within the tumor microenvironment (99, 100). Finally, HBx induces epithelial-mesenchymal transition (EMT) in HCC cells, facilitating their invasion and metastasis. By elucidating these intricate immunosuppressive mechanisms, we can identify potential therapeutic targets to disrupt cytokine signaling, reprogram neutrophils, target the HBx-TLR4-NF-κB axis, and reverse EMT, thereby liberating the immune system and enhancing anti-tumor responses in HBV-HCC.

#### 3.4.3 Invasion and metastasis

The tumor microenvironment (TME) plays a critical role in supporting tumor cell invasion and metastasis. Tumor-associated macrophages (TAMs) are a major component of the TME and exert significant influence on these processes, facilitating both invasion and metastasis. M2-polarized TAMs significantly contribute to cancer cell invasion and metastasis through various mechanisms, including the secretion of potent proteinases such as matrix metalloproteinases (MMPs) -2, -9, and cathepsins. These enzymes degrade extracellular matrix components, facilitating tumor cell migration and dissemination (101). Furthermore, M2-polarized TAMs contribute to cancer cell invasion and metastasis by inducing the epithelial-mesenchymal transition (EMT). Notably, these M2-TAMs have been shown to secrete epidermal growth factor (EGF). This potent pro-invasive factor can trigger EMT in cancer cells through activation of the EGFR-ERK signaling pathway (102). Macrophages are implicated in the EMT process through the secretion of diverse soluble mediators, including IL-1β, IL-8, TNF-α, and TGF-β (103). Immunohistochemical analysis of clinical HCC specimens demonstrated a spatial co-localization of EMT hotpots, particularly at the periphery of tumor nests, with abundant infiltration of TAMs (104, 105). TAMs are implicated in promoting cancer cell EMT through the paracrine activation of the nuclear factor (erythroid-derived 2)-like 2 (Nrf2) pathway and subsequent upregulation of VEGF (106). Additionally, HCC cells secrete Wnt ligands, which promote the polarization of TAMs towards an M2 phenotype, which contributes to tumor growth, metastasis, and immunosuppression (107). A recent study has identified microRNAs (miRNAs), particularly miR-98, as potential regulators of cancer cell invasion in HCC and suggests that miR-98 promotes the polarization of macrophages from the tumor-promoting M2 phenotype to the anti-tumor M1 phenotype (108). Interestingly, long non-coding RNAs (lncRNAs) also appear to play a crucial role in macrophage-mediated tumorigenesis in HCC. For instance, lncRNA COX-2 has been shown to suppress immune evasion and metastasis by inhibiting macrophage polarization towards the M2 phenotype (109). Collectively, these findings highlight the multifaceted interplay between macrophages, EMT, and miRNAs/lncRNAs in HCC progression. Targeting these interactions holds promise for developing novel therapeutic strategies to impede tumor growth, metastasis, and immunosuppression.

#### 3.4.5 Angiogenesis

TAMs orchestrate a potent pro-angiogenic cytokine milieu, with VEGF emerging as a key conductor. Profusely secreted by TAMs within the HBV-HCC microenvironment, VEGF acts as a potent chemoattractant and mitogen for endothelial cells, ultimately driving their assembly into functional neovasculature. IL-1 signaling exerts pro-angiogenic effects by amplifying the expression of VEGF and other angiogenic factors through the MAPK/JNK pathways (110). Additionally, HIF-1α upregulation, triggered by IFN-γ in MSCs, acts as a key mediator of increased VEGF expression, leading to enhanced tumor angiogenesis (111). Moreover, IL-8 triggers HCC metastasis and colonization via PKC/ERK1/2-mediated upregulation of matrix metalloproteinase 9 (MMP9) (112). Previously, TNF-α was implicated in suppressing tumor angiogenesis; however, recent investigations suggest a more nuanced role, revealing its potential for pro-angiogenic activity within the tumor microenvironment (113). The downregulation of miR-325-3p and subsequent upregulation of CXCL17 in HCC tissues not only directly promote angiogenesis but also align with the observed ability of TAMs to manipulate the chemokine landscape and facilitate blood vessel formation (114, 115). This convergence of independent mechanisms (miR-325-3p/CXCL17 and TAM activity) suggests potential synergy in promoting HCC angiogenesis, warranting further investigation. Targeting both pathways, via miR-325-3p upregulation or CXCL17/TAM inhibition, could offer novel therapeutic strategies to disrupt HCC neovascularization and impede tumor growth.

The cunning HBV X protein (HBx) emerges as a master manipulator in TAM-mediated angiogenesis (116). Interacting with TAMs via the Toll-like receptor 4 (TLR4) pathway, HBx activates the NF-κB signaling cascade, culminating in heightened VEGF production and amplification of the pro-angiogenic symphony (117). Furthermore, the hepatitis B virus preS2 domain has been shown to promote angiogenesis in HCC by transactivating the VEGFA promoter (118). In the intricate dance of HBV-related HCC, the viral X protein (HBx) emerges as a multifaceted conductor, wielding its influence over a diverse repertoire of cellular processes. Notably, HBx exhibits a distinct talent for regulating the expression of key cytokines and chemokines, including IL-6, IL-18, and CXCL12 (119). These factors exert profound downstream effects, orchestrating a symphony of cellular events critical for tumor progression. CXCL12, a key player in the angiogenic ballet, guides the recruitment and assembly of endothelial cells, paving the way for the intricate vasculature that fuels tumor growth and metastasis.

In the hypoxic tumor microenvironment, TAM-driven angiogenesis is further fueled as hypoxia promotes TAM infiltration and polarization towards a pro-angiogenic phenotype (120). Hypoxia-inducible factor-1 (HIF-1), a key regulator of cellular adaptation to low oxygen, upregulates pro-angiogenic factors in both TAMs and tumor cells, establishing a cyclic relationship of hypoxia-driven angiogenesis (121).

## 4 CD8+ T cells as adaptive immune response against HBV-related HCC

### 4.1 CD8+ T cells in controlling HBV infection

CD8+ T cells, integral effectors of the adaptive immune response, play a pivotal role in the control of HBV infection, a condition intricately linked to the development of HCC (14). Their arsenal features potent effector mechanisms: Recognizing HBV-infected hepatocytes through viral antigen presentation on MHC-I molecules, CD8+ T cells unleash a lethal barrage of granzymes and perforin, effectively lysing the virus-harboring cells (122). The central memory T cells (TCM), on the other hand, reside in secondary lymphoid tissues and serve as a long-lived reservoir of HBV-specific memory, enabling a swift and robust recall response upon re-exposure to the virus. TEFF cells, with a shorter lifespan and limited proliferative capacity, are the most potent cytotoxic cells but also exhibit increased susceptibility to apoptosis (123). Beyond direct killing, CD8+ T cells orchestrate the immune response through cytokine secretion. IFN-γ restricts viral replication, while IL-2 fuels further T cell proliferation and recruitment. Additionally, chemokines released by CD8+ T cells and other immune cells attract additional immune cells to the site of infection, amplifying the antiviral response (124). The efficacy of T cell response is contingent upon highly specialized TCRs, meticulously honed to recognize specific HBV-derived peptides presented on MHC-I molecules. The presence of divergent viral genotypes and immune escape mechanisms necessitate a diverse repertoire of TCRs for broad-spectrum viral control (125). Therefore, CD8+ T cells stand as a frontline defense against HBV, wielding a sophisticated toolbox to quell viral replication and maintain immune homeostasis. However, within the hostile HCC microenvironment, their effectiveness can be significantly compromised, setting the stage for the next chapters of the narrative.

During acute and chronic HBV infection, HBeAg is frequently expressed (126) and has been widely regarded as a key immunomodulator for promoting host innate and adaptive immune tolerance during chronic HBV infection through diverse mechanisms, such as down-regulation of toll-like receptor (TLR) expression, modulation of macrophage function, and induction of MDSC activation (127). Previously findings demonstrate that liver sinusoidal endothelial cells (LSECs) also switch from a tolerogenic state to an immunogenic state upon stimulation with HBV e antigen (HBeAg) (128). Moreover, within inflamed hepatic environments, LSECs undergo a remarkable phenotypic transition from tolerogenic facilitators to potent immunogenic players, triggering robust activation of cytotoxic effector CD8+ T cells, and orchestrating a potent anti-inflammatory response (129, 130). Recent findings provide compelling evidence that HBeAg acts not only as an immunosuppressor but also as a driver of LSEC maturation, thereby potentiating anti-HBV CTL function within the intrahepatic immune microenvironment. Co-culture of activated effector T cells with HBeAg-exposed liver sinusoidal endothelial cells (LSECs) results in significantly elevated IFN-γ production compared to T cells co-cultured with control LSECs. This suggests that HBeAg enhances the antigen-presenting function of LSECs, facilitating robust T-cell activation during HBV infection. LSEC-mediated T cell activation relies on upregulated TNFα and IL27 expression within these non-lymphoid players (127). Furthermore, *In vivo* studies of an HBV-replicating mouse model, where TNFα blockade significantly impairs HBV-specific CD8+ T cell immunity and delays viral clearance (128). These findings collectively paint a fascinating picture of the dynamic interplay between HBeAg, LSECs, and CD8+ T cells in HBV infection. HBeAg, once considered solely a suppressor, exhibits the unexpected ability to activate LSECs and ignite robust anti-viral CTL responses through TNFα and IL27-mediated pathways. Understanding this nuanced dialogue within the hepatic microenvironment holds immense potential for developing novel therapeutic strategies aimed at boosting anti-HBV immunity and achieving viral clearance.

### 4.2 CD8+ T cells and antitumor role

CD8+ T cells play a crucial role in the antitumor immunity against HCC arising from hepatitis B virus (HBV) infection. Cytotoxic T lymphocytes (CTLs), identified by their surface expression of CD8 co-receptor, are central players in the antitumor immune response. As the most prevalent T cell subset in many tumors, CD8+ T lymphocyte infiltration (TIL) levels have emerged as a significant prognostic factor, with high infiltration often correlating with improved clinical outcomes (131). The functional characteristics of CD8+ T cells, particularly cytokine secretion (IL-2, TNF-α, IFN-γ) and cytolytic granule presence (granzyme B), are intricately tied to their anti-tumor activity (132). Previous studies in colorectal cancer and lymphoma have revealed a positive correlation between CD11c upregulation on tumor-infiltrating CD8+ T cells and their tumoricidal efficacy. This suggests that CD11c expression may serve as a valuable marker for identifying potent cytotoxic T lymphocytes within the tumor microenvironment, potentially predicting the effectiveness of anti-tumor immunotherapy (133). Recently, a study suggests that CD11c+CD8 T cells in HBV-related HCC possess anti-tumor potential and that GB+CD11c+CD+ T cells are associated with disease progression in patients (134). Unraveling the complex story of CD11c and granzyme B within HBV-related HCC has the potential to identify potential therapeutic targets, and decipher distinct roles in HBV-related HCC progression and prognostic tools, pushing the tide toward improved clinical outcomes for patients.

### 4.3 CD8+ T cell exhaustion and inhibitory receptor (IR)

**Figure 3:**
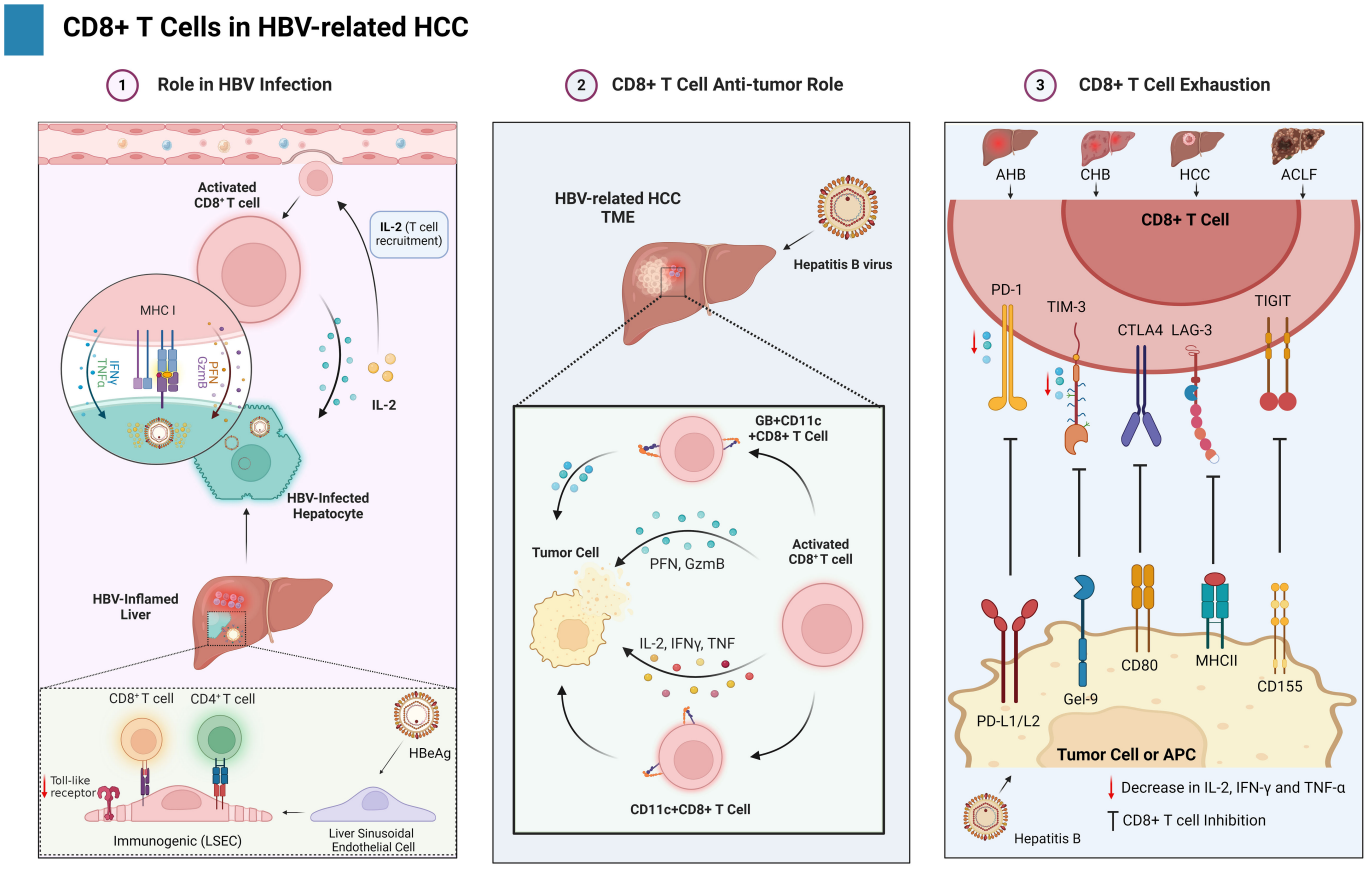
Illustrates the dual nature of CD8+ T cells in the context of HBV-HCC. It is establishing a balance between viral management, anticancer activity, and exhaustion against cancer.

The complex process of CD8+ T cell exhaustion presents a powerful obstacle to effective viral control and tumor immunity in HBV-related HCC. This dysfunctional state arises from a complex interplay of mechanisms, encompassing chronic antigen exposure-induced dysregulation of signaling pathways and transcriptional reprogramming, upregulation of co-inhibitory receptors and their ligands, altered cytokine profiles with both reduced effector and persistent pro-inflammatory responses, and metabolic reprogramming favoring inefficient glycolysis over robust oxidative phosphorylation as depicted in **Figure 3** (135, 136). Understanding these intricate mechanisms is crucial for devising effective therapeutic strategies aimed at reinvigorating exhausted T cells and unleashing their anti-tumor potential in HBV-associated HCC.

Persistent antigenic stimulation in chronic infections and tumor microenvironments triggers molecular and cellular pathways that culminate in a dysfunctional state of T cells known as exhaustion (137, 138). Various viral pathogens, including HCV and HBV, exhibit a well-documented correlation with T lymphocyte exhaustion (139). CD8+ T cell exhaustion, manifested by functional impairment and quantitative reduction, serves as a key driver of CHB progression (140). Exhausted CD8+ T cell subsets exhibit a graded spectrum of phenotypic and functional alterations depending on their differentiation state (138). Inhibitory receptors (IRs) slowly rise in T cells, indicating functional exhaustion (14). The coordinated upregulation of multiple inhibitory receptors (IRs), including programmed cell death protein-1 (PD-1), T cell immunoglobulin and mucin domain-3 (TIM-3), cytotoxic T-lymphocyte-associated protein 4 (CTLA-4), lymphocyte-activation gene 3 (LAG-3), T cell immunoreceptor with Ig and ITIM domains (TIGIT), CD244 (2B4), and CD160, constitutes a defining characteristic of human CD8+ and CD4+ T cell exhaustion (23, 141–143). Two independent studies have convincingly established a link between HBV-specific CD8+ T cells and enhanced IR expression, suggesting a potential mechanism for T cell dysfunction in chronic HBV infection (144, 145). Multiple lines of evidence suggest a significant upregulation of PD-1 expression on CD8+ T cells in all clinical phases of HBV infection, encompassing AHB, CHB, HCC, and acute-on-chronic liver failure (ACLF) (146–148). A novel study demonstrates that CD8+ T cells in individuals with CHB exhibit a distinct phenotype characterized by high levels of PD-1, TIM3, and CTLA4 expression (140). Furthermore, the involvement of additional inhibitory pathways, encompassing immunoregulatory cells, immunosuppressive cytokines, and inhibitory mediators, in T cell exhaustion needs further investigation, as their precise roles remain less well-characterized. Understanding the complex interplay between T cell exhaustion, immune checkpoints, and ICI therapy in HBV-related HCC holds immense promise for developing more effective and personalized treatment strategies. This battlefield may be fraught with challenges, but armed with knowledge and advanced therapies, we may yet turn the tide against this formidable foe.

Inhibitory receptors other than PD-1 has also significant role in HBV-related HCC CD8+ T cell exhaustion such as (TIM-3, CTLA-4, LAG-3, and TIGIT). TIM-3 is recognized as a critical IR on CD8+ T cells. It binds to its ligand, Galectin-9, leading to T-cell dysfunction. Studies have consistently shown elevated TIM-3 expression on CD8+ T cells isolated from HBV-infected patients across various clinical phases, including CHB, AHB, LC, and HCC, compared to healthy controls (145, 149). This overexpression of TIM-3 on CD8+ T cells is functionally linked to their impairment, encompassing diminished proliferation, reduced cytokine production (IL-2, IFN-γ, and TNF-α), and heightened susceptibility to apoptosis (150). Notably, research suggests a correlation between TIM-3 expression and disease severity (151). Additionally, another study revealed a positive correlation between the percentage of TIM-3+CD8+ T cells and HBV DNA load, further supporting the potential role of TIM-3 in regulating the immune response during HBV infection (145). CTLA-4 (CD152) upregulation on HBV-specific CD8+ T cells has been implicated in increased expression of pro-apoptotic proteins, such as B-cell lymphoma-2 (BCL-2) interacting mediator of cell death (BIM), ultimately leading to T cell apoptosis (152, 153). LAG-3 (CD223) also represents a significant IR significantly expressed on CD8+ T cells in patients (141). Notably, downregulated LAG-3 expression has been linked to suppressed IFN-γ secretion from CD8+ T cells in CHB and HCC patients. Furthermore, TIGIT, another inhibitory receptor (IR) belonging to the immunoglobulin superfamily, has been implicated in CD8+ T cell dysfunction in HBV infection (154). Co-expression of PD-1 and TIGIT on these cells has been associated with an exhausted phenotype, characterized by an increased apoptosis rate, expression of exhaustion-related TFs (Eomes/T-bet), and decreased cytokine production (155). Notably, TAMs have been shown to express Nectin (ligand) and contribute to the suppression of CD8+ T cells in HCC patients.

## 5 Interplay between TAMs and CD8+ T cells in HBV-related HCC

**Figure 4:**
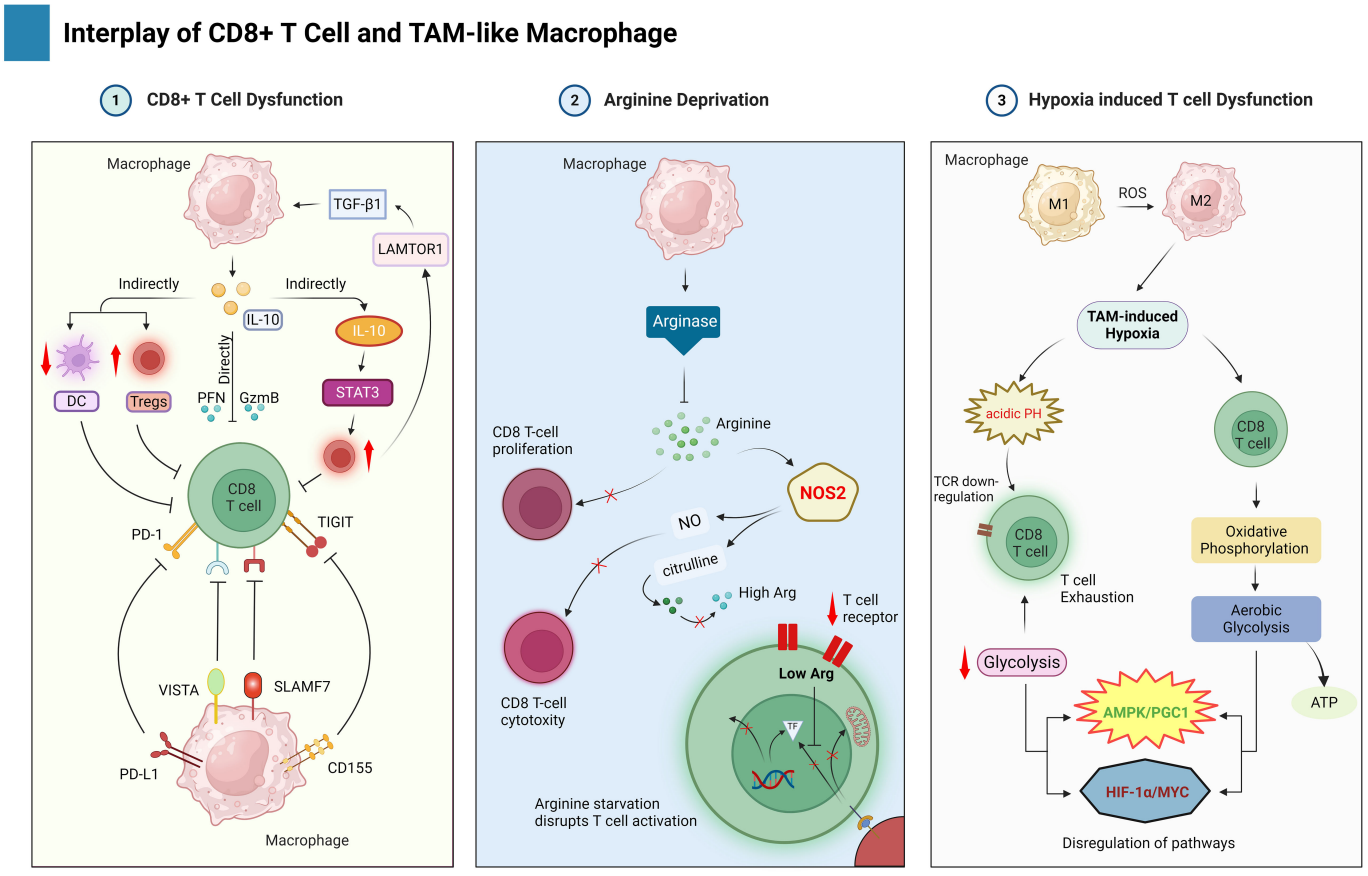
This figure highlights the interplay between TAMs and CD8+ T cells in the TME of HBV-related HCC. TAMs suppress CD8+ T cell function by several mechanisms such as direct and indirect macrophage events, arginine deprivation, and hypoxia.

The complex interplay between TAMs and CD8+ T in HBV-related HCC cells significantly shapes the TME, impacting disease progression and therapeutic outcomes. For patients with HCC, the secret to unlocking innovative immunotherapies lies in the intricate dance between immune activation and suppression as shown in **Figure 4**.

### 5.1 TAM-mediated CD8+ T cell dysfunction through surface molecules

TAMs often exhibit a pro-tumorigenic M2-like phenotype, characterized by the production of immunosuppressive cytokines and enzymes that hinder the antitumor activity of CD8+ T cells. IL-10 directly suppresses the activation and function of CD8+ T cells, downregulating their effector functions and cytokine production (156). IL-10 exerts a dual immunosuppressive effect: directly repressing genes encoding cytotoxic mediators like perforin, granzyme, and cytotoxins in CD8+ T cells, and indirectly inhibiting their activation by stimulating the expansion of regulatory T cells (Tregs) and suppressing the antigen-presenting functions of dendritic cells (DCs) (157, 158). Furthermore, TAMs themselves are influenced by signaling pathways like the phosphorylation of LAMTOR1, which interacts with Exo70 and other components to enhance the secretion of TGF-β1, which secret fuels a positive feedback loop, promoting TAM polarization towards a more immunosuppressive phenotype, marked by an increase in Tregs and a decrease in CD8+ T cells within the HCC tumor microenvironment (159). On the other hand, a potential counterpoint to this immunosuppression exists. The MAGE-A3 protein can act as a tumor antigen, presented by mature DCs, which can stimulate the maturation of CD8+ cytotoxic T lymphocytes (CTLs) specific for MAGE-A3. These MAGE-A3-specific CD8+ CTLs then possess the ability to directly kill HCC tumor cells, offering a potential avenue for immunotherapy. Further studies are needed to explore how to manipulate TAM polarization and leverage the anti-tumor potential of MAGE-A3-specific CD8+ CTLs for the development of novel therapeutic strategies in HCC.

TAMs can directly inhibit CD8+ T cell activity by expressing inhibitory ligands that bind to receptors on T cells, essentially putting the brakes on their immune response. TAMs emerge as the dominant PD-L1 source and immune modulators in tumors (160). TAM-derived PD-L1 expression significantly impacts CD8+ T cell function and response to anti-PD1/PDL1 therapy (161). Intriguingly, the TME, especially in highly immune-active HBV-related HCC tumors, appears to dynamically regulate TAM PD-L1 expression by several stimuli including cytokines like IL-10 and IL-27, signaling pathways such as TGF-β/PKM2, and classical M1 macrophage activation involving TNF-α/NFkB and MAPK pathways (74, 162). These stimuli often activate STAT1 and STAT3 signaling pathways, with STAT3 playing a particularly role in activation of IL-10, which further promote the expression of PD-L1 in Tregs and TAMs (163). Interestingly, TAMs have also been shown to take up tumor cell-derived PD-L1 for further expression (164). Beyond PD-L1, TAMs express novel checkpoint molecules like SLAMF7 and VISTA/PSGL1, potentially offering targets to modulate their immunomodulatory functions and drive T-cell exhaustion (165). CLEVER-1 and B7-H4 expression specifically marks suppressive TAMs, correlating with impaired cytokine production and dysfunctional T-cell activity (166). Targeting these various pathways offers promising avenues for therapeutic interventions to enhance anti-tumor immunity.

TAMs can express ligands for TIGIT, an inhibitory receptor expressed on CD8+ T cells, its interaction with ligands can lead to T cell exhaustion in various cancers including colorectal cancer (167). Additionally, the expression of TIGIT on CD8+ T cells is associated with pathogenesis and progression in patients with HBV-related HCC (155). These findings collectively support the idea that interactions between TIGIT and its ligands, which TAMs can express contribute to T cell exhaustion.

### 5.2 TAM-mediated arginine deprivation

Arginine, an essential amino acid, fuels critical functions in CD8+ T cells, impacting their proliferation, activation, and cytotoxic capacity (168). In the context of HBV-associated HCC, this vital resource becomes scarce due to a complex interplay within the tumor microenvironment. TAMs, abundant in HCC, express arginase, an enzyme that depletes arginine. This depletion directly hinders CD8+ T cell proliferation as they rely on arginine for metabolic processes (169). Furthermore, arginine is a substrate for nitric oxide (NO) production, a crucial molecule in CD8+ T cell-mediated cytotoxicity. Arginine depletion may also indirectly affect immune checkpoint regulation, potentially influencing molecules like PD-1/PD-L1 that modulate CD8+ T cell activity (170). In essence, arginase-1 acts as a competitor to nitric oxide synthase (NOS) for the limited pool of L-arginine, creating a microenvironment that starves CD8+ T cells of this essential amino acid and dampens their anti-tumor potential (171). Beyond starvation, arginine depletion exerts a multifaceted detrimental effect on CD8+ T cells. Studies have demonstrated its capacity to hinder the production of IL-2 and subsequent proliferation upon TCR stimulation, a critical hallmark of T-cell activation (172, 173). This functional impairment is thought to be partially mediated by the downregulation of CD3ζ, a proximal signaling molecule within the TCR pathway. Furthermore, chronic exposure to high levels of HBV viremia exacerbates this dysfunction, driving CD8+ T cells toward an exhausted state (174). This exhaustion manifests as a terminally differentiated phenotype and shortened telomeres, ultimately compromising their anti-tumor potential. These observations suggest a therapeutic avenue: strategies to replenish arginine or modulate its metabolism could bolster CD8+ T cell responses in HBV-HCC. This might involve targeting TAMs to curb their arginase expression or exploring methods to limit other arginine-depleting mechanisms, ultimately enhancing antitumor immunity.

### 5.3 TAM-mediated hypoxia and its impact on CD8+ T cell immunity

High oxygen consumption in oxidative phosphorylation can induce hypoxia in the TME, which inhibits the function of T cells in multiple ways (175). Notably, TAM-mediated hypoxia in HBV-HCC TME directly inhibits CD8+ T cell function, particularly by promoting prolonged interactions that lead to exhaustion. Additionally, hypoxia induces CD39 expression on exhausted T cells, leading to the generation of immunosuppressive adenosine (176). Furthermore, the acidic pH of a hypoxic TME has been shown to downregulate the expression of TCR and IL-2Ra, consequently inhibiting T cell function (177). Interestingly, this inhibition can be reversed using proton pump inhibitors. Reactive oxygen species (ROS) represent one of several factors known to modulate the functional phenotypes of macrophages, such as the M1 and M2 polarization states (178). Following activation, T cells undergo a metabolic shift from oxidative phosphorylation to aerobic glycolysis. This metabolic reprogramming prioritizes the generation of ATP to meet their heightened energy demands while minimizing the production of ROS. However, in response to the metabolic constraints imposed by the TME, activated T cells exhibit a metabolic shift. They preferentially activate the AMPK/PGC1 pathway while suppressing the HIF-1α and MYC pathways, leading to a downregulation of glycolysis (179, 180). This metabolic reprogramming, however, has detrimental consequences. The subsequent overproduction of ROS and resultant mitochondrial stress ultimately culminate in T-cell exhaustion (181). One approach to target TAMs in HBV-related HCC is through the use of dimethyl formamide (DMF), which can significantly inhibit the tumor invasion-promoting ability of TAMs in the co-culture system (182). DMF functions by reducing ROS production in TAMs, leading to a decrease in tumor metastasis and an increase in T-cell infiltration into the tumor (182).

## 6 Therapeutic strategies

### 6.1 CD8+ T cells in HBV-related HCC

In the context of HBV-related HCC, targeting CD8+ T cells represent a promising therapeutic strategy, with a particular focus on immune checkpoint inhibitors (ICIs). Checkpoint blockade, particularly targeting the PD-1/PD-L1 pathway, represents a pivotal immunotherapeutic strategy in chronic viral infections (183). Multiple studies have demonstrated that blocking this inhibitory interaction on CD8+ T cells restore their function, as evidenced by increased proliferation, cytotoxicity (perforin, granzyme), and secretion of antiviral cytokines such as IL-2, IL-12p70, IFN-γ, TNF-α, IL-6 as shown **Table 1** (184, 185). Additionally, PD-1/PD-L1 blockade reduces IL-10 production and enhances T-cell sensitivity to apoptosis (186). However, Tang et al. observed no significant impact of PD-1 blockade on CD8+ T cell function in CHB patients (187). Similarly, Gehring et al. demonstrated that PD-1/PD-L1 blockade increased the number of CD8+ T cells in HCC patients, but did not restore IFN-γ and TNF-α production (188). Notably, CD8+ T cell exhaustion levels and frequencies were a major determinant of responsiveness to PD-1 blockade; also, PD-1 inhibition is unable to restore the higher frequencies of severely exhausted CD8+ T cell activity (189). Conversely, several studies have shown that blocking PD-1 together with other inhibitory receptors (IRs) such as TIM-3, LAG-3, CTLA-4, BTLA, and CD244 or stimulating CD137 (CD137L), results in more effective effects compared to blocking a single receptor (142, 190–192). Regarding this, Bengsch et al. have demonstrated that the reaction to blocking antibodies is highly variable and that PD-1 blocking is linked to the strongest effects on T cell responses when compared to other IRs like CTLA-4, TIM-3, 2B4, and BTLA blockage due to PD-1’s dominant expression on T cells (193). Remarkably, as expected, PD-1/PD-L1 inhibition reduced the activity of CD8+CXCR5+ T cells with exhausted resistance (194). The evidence suggests that combination of PD-1 blockade with targeting additional inhibitory molecules for further investigation is a potential approach to improve exhausted T cell function.

**Table 1:** Advances in checkpoint inhibitor in HBV-HCC.

Therapeutic Strategy	Target Mechanism	Potential Benefit in HBV-related HCC	Reference
*PD-1 Checkpoint Inhibitor* *CD244 Checkpoint Inhibitor* *PD-1/CD137 Combination Therapy* *BTLA Checkpoint Inhibitor* *PD-1 Blockade with Intermediate T cell Differentiation*	Blocks PD-1 receptor on CD8+ T cells, promoting T cell activation Blocks CD244 receptor on CD8+ T cells, promoting T cell activation and restoration independent of the PD-1 pathway Blocks PD-1 and activates CD137 signaling on CD8+ T cells Blocks BTLA inhibitory receptor on CD8+ T cells PD-1 blockade in patients with HBV-specific CD8+ T cells exhibiting intermediate differentiation	Enhanced anti-tumor T cell response against HBV-infected hepatocytes Restored T cell function and potential for enhanced anti-tumor T cell response against HBV-infected hepatocytes Enhanced activation and responses of intrahepatic HBV-specific T cells Restoration of antiviral T cell responses and improved T cell function in HBV infection Potential for enhanced therapeutic response	(184) (192) (186) (209) (193)
*TIM-3 Checkpoint Inhibitor* *TIM-3 and PD-1 Combination Blockade* *Galectin-9 Blockade*	Blocks TIM-3 receptor on CD8+ T cells, promoting T cell proliferation and antiviral cytokine secretion Blocks TIM-3 and PD-1 receptors on CD8+ T cells Blocks Gal-9 on NK cells, preventing interaction with TIM-3 on CD8+ T cells	Enhanced anti-tumor T cell response against HBV-infected hepatocytes Potentially greater restoration of functional antiviral responses compared to PD-1 blockade alone Potentially restores anti-tumor T cell function and enhances antiviral response	(197) (195) (150)

Similar to PD-1, TIM-3 is another inhibitory receptor expressed in T cells. Several studies have demonstrated that the inhibition of TIM-3 leads to the reversal of various functions of CD8+ T lymphocytes, including proliferation, cytokine generation, and cytotoxicity, while also reducing early apoptosis (195, 196). In CHB patients, an upregulation of Gal-9 expression in NK cells has been observed. This Gal-9 interacts with the T cell exhaustion marker, TIM-3, leading to the suppression of cytotoxic CD8+ T cell activity (150). Interestingly, Nebbia et al. investigated the relative effectiveness of blocking Tim-3 versus PD-1, another immune checkpoint receptor. Their findings demonstrated that blockade of the Tim-3 pathway exhibited superior therapeutic efficacy compared to PD-1 blockade (197). These results contrast with Bengsch et al. and Martin et al. who reported similar or greater efficacy of PD-1 blockade (193). Further research is needed to figure out the comparative efficacy of anti-TIM-3 versus anti-PD-1 blocking and to investigate any potential synergy between these methods when used in conjunction with other immunomodulatory strategies. The comprehensive review has the potential to enhance therapy regimens for better clinical outcomes in individuals with CHB.

### 6.2 TAMs in HBV-related HCC

TAMs, often displaying an immunosuppressive M2-like phenotype, contribute to establishment of an immune-tolerant microenvironment in HCC. Immune checkpoint inhibitors, such as those targeting PD-1 and its ligand PD-L1, have emerged as key modalities to disrupt TAM-mediated immunosuppression (198). Pharmacological inhibitors that block the PD-1/PD-L1 pathway have been demonstrated to increase T-cell cytotoxicity and activity, and dramatically slow the growth of HCC tumors (198). While PD-1/PD-L1 checkpoint blockade has shown promise, its effectiveness remains limited. Interestingly, research suggests that anti-PD-1 therapy not only reduces immunosuppression but also promotes M1 macrophage polarization, enhancing antitumor effects (199). Beyond PD-1/PD-L1, CD47 is another potential target, as its interaction with SIRPα on macrophages facilitates tumor escape (200). Blocking CD47 can reverse this immunosuppressive effect. Furthermore, M1 macrophage polarization strategies, such as Listeria-based vaccines, can work synergistically with PD-L1 blockade to re-sensitize T cells to immunotherapy (201). Adoptive cell therapy using M1-like CAR macrophages (CAR-Ms) offers an exciting avenue, demonstrating potent antitumor activity even in presence of immunosuppressive M2 macrophages (202). On the other hand, drug delivery systems like MC3 lipid nanoparticles can effectively target the liver and promote M1 polarization, reducing the side effects associated with systemic immunotherapy (203). As chronic HBV infection often contributes to CD8+ T cell exhaustion, combination therapies involving ICIs and agents that specifically target TAMs are being explored to synergistically enhance therapeutic efficacy. While challenges such as response durability and identifying optimal combination regimens remain, the targeting of TAMs and CD8+ T cells, particularly through immune checkpoint inhibition, represents a compelling avenue for advancing the treatment landscape of HBV-related HCC. These combined approaches highlight the potential of TAM manipulation as a cornerstone of effective HCC immunotherapy.

## 7 Challenges and future directions

The complex interplay between TAMs and CD8+ T cells within the HCC microenvironment, particularly in CHB infection, presents a significant barrier to effective immunotherapy. M2-like TAMs actively suppress antitumor immunity through immunosuppressive factors, hindering the function of CD8+ T cells, critical for HCC control. The secretion of immunosuppressive cytokines and expression of inhibitory molecules by TAMs can impede the functionality of CD8+ T cells, critical effectors in the antitumor immune response. Furthermore, chronic HBV infection exacerbates this by driving CD8+ T cell exhaustion (97). Overcoming these immune evasion mechanisms is crucial. Identifying reliable biomarkers that reflect TAM polarization, CD8+ T cell exhaustion, and the overall immune landscape holds immense potential. Such biomarkers could not only improve our understanding of the TAM-CD8+ T cell interplay but also pave the way for the development of personalized immunotherapies tailored to the unique immunological profile of each patient.

Reprogramming TAMs towards an antitumorigenic M1-like phenotype and overcoming CD8+ T cell exhaustion are crucial challenges in developing effective immunotherapies for HBV-related hepatocellular carcinoma (HCC). Studies have shown that the drug tadalafil (TA) inhibits M2 polarization and polyamine metabolism in TAMs, potentially reshaping the immunosuppressive tumor microenvironment (204). Additionally, the emergence of inhibitory receptors like PD-1 on CD8+ T cells contribute to exhaustion, particularly in CHB and HCC patients (23). Furthermore, the oncogenic reprogramming of HCC methionine recycling has been linked to T-cell exhaustion, suggesting that targeting tumor metabolism could enhance HCC immunity (205). Understanding the differential TME characteristics between true and *de novo* HCC recurrences can guide the development of tailored immunotherapy strategies (206). In HBV-related HCC, overcoming immune cell evasion involves targeting immunosuppressive mechanisms like LAG3, TIM3, and metabolic adaptations to enhance CD8 T cell response for improved outcomes. Studies highlight the role of the TME in HCC progression and immune evasion (21). Notably, MYC oncogene amplification in HCC leads to immune-checkpoint expression, hindering immune surveillance and response, which is reversed by combined PDL1 and CTLA4 blockade (207). Additionally, the interaction between CD3+C1q+TAM and CD8+ CCL4+ T cells influence T cell immunity in HCC, suggesting potential strategies to regulate the immunosuppressive TME (21). Furthermore, targeting the thioredoxin reductase (Trx) system with butaselen (an organoselenium-containing compound) promotes NK and T cell activity in the TME, inhibiting the immune escape of HCC cells and enhancing the efficacy of PD-1 blockade (208). These findings collectively emphasize the importance of targeting immunosuppressive pathways and metabolic adaptations to improve CD8 T cell responses in HBV-related HCC.

Deciphering the intricate interplay between TAMs and CD8+ T cells within the HBV-related HCC microenvironment is crucial for overcoming the current hurdles in immunotherapy. Future research should prioritize unraveling the molecular pathways governing TAM-CD8+ T cell crosstalk through integration of high-throughput omics technologies and advanced molecular profiling. This deeper understanding will facilitate the identification of specific targets to disrupt immunosuppressive signals from TAMs and develop strategies to rejuvenate exhausted CD8+ T cells. Furthermore, the development of non-invasive imaging techniques can provide dynamic insights into the spatial and temporal aspects of this interplay, aiding in the discovery of novel biomarkers for patient stratification and the design of personalized therapeutic approaches tailored to the unique immunological landscape of each patient. By addressing these knowledge gaps and fostering a multifaceted approach, researchers can pave the way for development of more effective immunotherapies to combat HBV-related HCC.

## 8 Conclusion

Immunotherapy has transformed the treatment of certain cancers, but its effectiveness in hepatocellular carcinoma (HCC) is limited by the immunosuppressive tumor microenvironment. CD8+ T cells, which are key immune system effector cells, play an important role in antitumor immunity. However, in HCC, their activity is frequently restricted by an immunosuppressive microenvironment driven by tumor-associated macrophages (TAMs).

Manipulating TAM polarization towards an antitumoral phenotype, reducing immunosuppressive TAMs, or enhancing CD8+ T cell function by disrupting TAM-mediated suppression shows potential for enhancing HCC treatment strategies. Advancements in single-cell spatial technologies offer exciting possibilities to further investigate the intricate interplay between CD8+ T cells and TAMs. By analyzing the transcriptional profiles of these cell types within their precise locations in the tumor microenvironment, researchers can gain unprecedented insights into the complicated communication and regulation occurring at the cellular level. This knowledge can help to develop more targeted and effective HCC immunotherapies.
